# 3,3-Dimethyl-9-phenyl-3,4-dihydro­acridin-1(2*H*)-one

**DOI:** 10.1107/S1600536808022083

**Published:** 2008-07-19

**Authors:** Hosein Ghorbani, Ayoob Bazgir

**Affiliations:** a Department of Chemistry, Islamic Azad University, Dorood Branch, Dorood 688173551, Iran

## Abstract

In the mol­ecule of the title compound, C_21_H_19_NO, the terminal saturated six-membered ring of the dihydro­acridine unit adopts an envelope conformation, while the other two fused rings are nearly coplanar, with a dihedral angle of 2.61 (3)°. The coplanar ring system is oriented with respect to the phenyl ring at a dihedral angle of 74.58 (3)°. In the crystal structure, there is a C—H⋯π contact between the central ring of the dihydro­acridine system and the phenyl ring and a π–π contact between the two central rings [centroid–centroid distance = 3.809 (1) Å].

## Related literature

For general background, see: Kalluraya & Sreenivasa (1998 ▶); Doube *et al.* (1998 ▶); Maguire *et al.* (1994 ▶). For bond-length data, see: Allen *et al.* (1987 ▶).
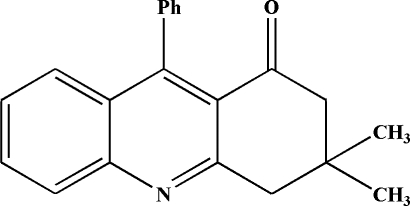


## Experimental

### 

#### Crystal data

C_21_H_19_NO
*M* *_r_* = 301.37
Monoclinic, 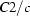

*a* = 16.341 (3) Å
*b* = 11.3889 (18) Å
*c* = 18.772 (4) Å
β = 110.386 (14)°
*V* = 3274.8 (10) Å^3^
*Z* = 8
Mo *K*α radiation
μ = 0.07 mm^−1^
*T* = 298 (2) K
0.33 × 0.22 × 0.1 mm

#### Data collection

Stoe IPDSII diffractometer
Absorption correction: numerical (*X-SHAPE*; Stoe & Cie, 2005 ▶) *T*_min_ = 0.980, *T*_max_ = 0.990
11268 measured reflections
3882 independent reflections
3032 reflections with *I* > 2σ(*I*)
*R*_int_ = 0.044

#### Refinement

*R*[*F* ^2^ > 2σ(*F* ^2^)] = 0.061
*wR*(*F* ^2^) = 0.143
*S* = 1.10
3882 reflections
208 parameters
H-atom parameters constrained
Δρ_max_ = 0.24 e Å^−3^
Δρ_min_ = −0.26 e Å^−3^

### 

Data collection: *X-AREA* (Stoe & Cie, 2005 ▶); cell refinement: *X-AREA*; data reduction: *X-RED* (Stoe & Cie, 2005 ▶); program(s) used to solve structure: *SHELXS97* (Sheldrick, 2008 ▶); program(s) used to refine structure: *SHELXL97* (Sheldrick, 2008 ▶); molecular graphics: *ORTEP-3 for Windows* (Farrugia, 1997 ▶); software used to prepare material for publication: *WinGX* (Farrugia, 1999 ▶).

## Supplementary Material

Crystal structure: contains datablocks global, I. DOI: 10.1107/S1600536808022083/hk2495sup1.cif

Structure factors: contains datablocks I. DOI: 10.1107/S1600536808022083/hk2495Isup2.hkl

Additional supplementary materials:  crystallographic information; 3D view; checkCIF report

## Figures and Tables

**Table 1:** Hydrogen-bond geometry (Å, °) *Cg*1 is the centroid of the N1/C7/C8/C15/C20/C21 ring.

*D*—H⋯*A*	*D*—H	H⋯*A*	*D*⋯*A*	*D*—H⋯*A*
C11—H11⋯*Cg*1^i^	0.93	3.20	3.814 (3)	126

Symmetry code: (i) 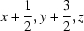.

**Fig. 1.**
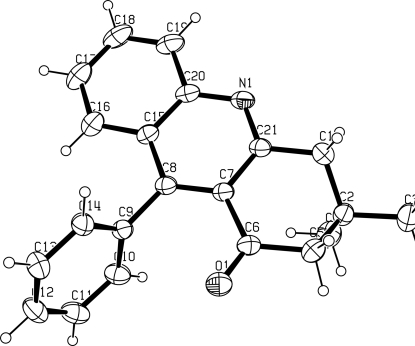
The molecular structure of the title molecule, with the atom-numbering scheme. Displacement ellipsoids are drawn at the 30% probability level.

## supplementary crystallographic information

### Comment

Recently, quinolines and their derivatives have received considerable attention, due to their wide range of therapeutic and biological properties. They have emerged as antimalarial, antiasthmatic, anti-inflamatory, antibacterial, anti- hypertensive and tyrosine kinase PDGF-RTK inhibiting agents. Moreover, poly- quinolines are found to undergo hierarchical self-assembly into a variety of nano and *meso* structures with enhanced electronic and photonic functions (Kalluraya & Sreenivasa, 1998; Doube *et al.*,  1998; Maguire *et al.*,  1994). We report herein the synthesis and crystal structure of the title compound.

In the molecule of the title compound (Fig. 1), the bond lengths (Allen *et al.*,  1987) and angles are within normal ranges. Ring A (C1/C2/C5-C7/C21) adopts envelope conformation, with C2 atom displaced by -0.683 (3) Å from the plane of the other ring atoms. Rings B (N1/C7/C8/C15/C20/C21), C (C15-C20) and D (C9-C14) are, of course, planar and they are oriented at dihedral angles of B/C = 2.61 (3)°, B/D = 74.17 (3)° and C/D = 75.01 (3)°. So, rings B and C are nearly coplanar. The coplanar ring system is oriented with respect to the phenyl ring D at a dihedral angle of 74.58 (3)°.

In the crystal structure, a C—H···π contact (Table 1) between rings B and D and a π—π contact between the symmetry related B rings Cg1···Cg1^i^ [symmetry code: (i) 1/2 - x, 3/2 - y, - z, where Cg1 is the centroid of ring B] may stabilize the structure, with centroid-centroid distance of 3.809 (1) Å.

### Experimental

For the preparation of the title compound, a mixture of 5,5-dimethylcyclohexane -1,3-dione (1 mmol), (2-aminophenyl)(phenyl)methanone (1 mmol) and benzyl tri- ethyl ammonium chloride (0.1 g) in water (5 ml) was stirred at reflux for 5 h. After completion of reaction (monitored by TLC) the reaction mixture was filtered and the precipitate washed with water (15 ml), and then recrystallized from EtOH/water (1:2) to afford the pure product (yield; 0.195 g, 65%).

### Refinement

H atoms were positioned geometrically, with C-H = 0.93, 0.97 and 0.96 Å for aromatic, methylene and methyl H, respectively, and constrained to ride on their parent atoms with U_iso_(H) = 1.2U_eq_(C).

### Crystal data

**Table:**

C_21_H_19_NO	*F*_000_ = 1280
*M**_r_* = 301.37	*D*_x_ = 1.222 Mg m^−^^3^
Monoclinic, *C*2/*c*	Mo *K*α radiation λ = 0.71073 Å
Hall symbol: -C 2yc	Cell parameters from 1575 reflections
*a* = 16.341 (3) Å	θ = 2.2–28.0º
*b* = 11.3889 (18) Å	µ = 0.08 mm^−^^1^
*c* = 18.772 (4) Å	*T* = 298 (2) K
β = 110.386 (14)º	Block, colorless
*V* = 3274.8 (10) Å^3^	0.33 × 0.22 × 0.1 mm
*Z* = 8	

### Data collection

**Table:**

Stoe IPDSII diffractometer	*R*_int_ = 0.044
rotation method scans	θ_max_ = 28.0º
Absorption correction: numericalshape of crystal determined optically (X-SHAPE; Stoe & Cie, 2005)	θ_min_ = 2.2º
*T*_min_ = 0.980, *T*_max_ = 0.990	*h* = −21→21
11268 measured reflections	*k* = −14→15
3882 independent reflections	*l* = −24→17
3032 reflections with *I* > 2σ(*I*)	

### Refinement

**Table:**

Refinement on *F*^2^	H-atom parameters constrained
Least-squares matrix: full	*w* = 1/[σ^2^(*F*_o_^2^) + (0.0491*P*)^2^ + 1.8747*P*] where *P* = (*F*_o_^2^ + 2*F*_c_^2^)/3
*R*[*F*^2^ > 2σ(*F*^2^)] = 0.061	(Δ/σ)_max_ = 0.002
*wR*(*F*^2^) = 0.143	Δρ_max_ = 0.24 e Å^−^^3^
*S* = 1.10	Δρ_min_ = −0.25 e Å^−^^3^
3882 reflections	Extinction correction: none
208 parameters	

### Special details

**Table:**

Geometry. All e.s.d.'s (except the e.s.d. in the dihedral angle between two l.s. planes) are estimated using the full covariance matrix. The cell e.s.d.'s are taken into account individually in the estimation of e.s.d.'s in distances, angles and torsion angles; correlations between e.s.d.'s in cell parameters are only used when they are defined by crystal symmetry. An approximate (isotropic) treatment of cell e.s.d.'s is used for estimating e.s.d.'s involving l.s. planes.

### Fractional atomic coordinates and isotropic or equivalent isotropic displacement parameters (Å^2^)

**Table:**

	*x*	*y*	*z*	*U*_iso_*/*U*_eq_	
O1	0.81274 (12)	0.40574 (13)	−0.19111 (8)	0.0814 (5)	
N1	0.88041 (9)	0.16743 (13)	0.03136 (8)	0.0490 (3)	
C1	0.87761 (13)	0.08450 (15)	−0.08673 (11)	0.0533 (4)	
H1A	0.8206	0.048	−0.1079	0.064*	
H1B	0.9169	0.0277	−0.0535	0.064*	
C2	0.91044 (11)	0.11403 (15)	−0.15138 (10)	0.0494 (4)	
C3	0.90577 (17)	0.00459 (19)	−0.20010 (14)	0.0736 (6)	
H3A	0.9239	0.0244	−0.2421	0.088*	
H3B	0.8469	−0.0242	−0.219	0.088*	
H3C	0.9436	−0.055	−0.1698	0.088*	
C4	1.00438 (13)	0.1581 (2)	−0.12016 (13)	0.0681 (6)	
H4A	1.0074	0.2267	−0.0897	0.082*	
H4B	1.0236	0.1773	−0.1616	0.082*	
H4C	1.0413	0.0979	−0.0896	0.082*	
C5	0.85007 (13)	0.20855 (17)	−0.19917 (10)	0.0570 (4)	
H5A	0.8728	0.234	−0.238	0.068*	
H5B	0.7933	0.1736	−0.2249	0.068*	
C6	0.83780 (12)	0.31480 (15)	−0.15688 (9)	0.0493 (4)	
C7	0.85331 (10)	0.30320 (13)	−0.07363 (9)	0.0405 (3)	
C8	0.84960 (9)	0.39807 (14)	−0.02880 (9)	0.0411 (3)	
C9	0.83425 (10)	0.52170 (14)	−0.05688 (9)	0.0423 (3)	
C10	0.90001 (12)	0.58667 (17)	−0.06880 (11)	0.0550 (4)	
H10	0.9535	0.5517	−0.0623	0.066*	
C11	0.88648 (13)	0.70308 (17)	−0.09023 (12)	0.0635 (5)	
H11	0.9312	0.7465	−0.0973	0.076*	
C12	0.80695 (14)	0.75516 (16)	−0.10110 (11)	0.0626 (5)	
H12	0.7977	0.8333	−0.1162	0.075*	
C13	0.74135 (13)	0.69126 (16)	−0.08949 (12)	0.0595 (5)	
H13	0.6877	0.7264	−0.0968	0.071*	
C14	0.75471 (11)	0.57519 (15)	−0.06710 (10)	0.0496 (4)	
H14	0.7102	0.5327	−0.0589	0.06*	
C15	0.86231 (10)	0.37692 (15)	0.04941 (9)	0.0436 (4)	
C16	0.86349 (12)	0.46714 (19)	0.10169 (11)	0.0584 (5)	
H16	0.8566	0.5449	0.0856	0.07*	
C17	0.87457 (14)	0.4412 (2)	0.17538 (11)	0.0720 (6)	
H17	0.8753	0.5013	0.2091	0.086*	
C18	0.88480 (14)	0.3247 (2)	0.20068 (11)	0.0746 (6)	
H18	0.8914	0.308	0.2509	0.09*	
C19	0.88514 (13)	0.2360 (2)	0.15250 (11)	0.0640 (5)	
H19	0.8919	0.159	0.17	0.077*	
C20	0.87526 (10)	0.25947 (16)	0.07591 (9)	0.0464 (4)	
C21	0.87064 (10)	0.18891 (14)	−0.04023 (9)	0.0429 (4)	

### Atomic displacement parameters (Å^2^)

**Table:**

	*U*^11^	*U*^22^	*U*^33^	*U*^12^	*U*^13^	*U*^23^
O1	0.1371 (15)	0.0625 (8)	0.0472 (7)	0.0205 (9)	0.0353 (8)	0.0142 (7)
N1	0.0525 (8)	0.0514 (8)	0.0475 (8)	0.0020 (6)	0.0228 (6)	0.0132 (6)
C1	0.0635 (11)	0.0421 (8)	0.0588 (10)	−0.0016 (8)	0.0269 (9)	0.0013 (8)
C2	0.0542 (9)	0.0484 (9)	0.0504 (9)	−0.0015 (8)	0.0241 (8)	−0.0063 (7)
C3	0.0902 (15)	0.0633 (12)	0.0775 (14)	−0.0042 (11)	0.0420 (12)	−0.0184 (11)
C4	0.0554 (11)	0.0764 (13)	0.0784 (14)	−0.0041 (10)	0.0310 (10)	−0.0112 (11)
C5	0.0679 (11)	0.0613 (11)	0.0427 (9)	−0.0011 (9)	0.0205 (8)	−0.0042 (8)
C6	0.0590 (10)	0.0507 (9)	0.0398 (8)	0.0030 (8)	0.0192 (7)	0.0076 (7)
C7	0.0420 (8)	0.0436 (8)	0.0388 (8)	0.0000 (6)	0.0177 (6)	0.0050 (6)
C8	0.0386 (7)	0.0455 (8)	0.0421 (8)	0.0000 (6)	0.0178 (6)	0.0053 (7)
C9	0.0469 (8)	0.0433 (8)	0.0384 (8)	−0.0016 (7)	0.0170 (6)	0.0009 (6)
C10	0.0464 (9)	0.0595 (11)	0.0592 (11)	−0.0024 (8)	0.0187 (8)	0.0118 (9)
C11	0.0650 (12)	0.0596 (11)	0.0626 (12)	−0.0196 (9)	0.0179 (9)	0.0093 (9)
C12	0.0777 (13)	0.0381 (9)	0.0645 (12)	−0.0062 (9)	0.0152 (10)	0.0042 (8)
C13	0.0607 (11)	0.0460 (9)	0.0703 (12)	0.0065 (8)	0.0211 (9)	0.0007 (9)
C14	0.0509 (9)	0.0445 (9)	0.0579 (10)	−0.0011 (7)	0.0245 (8)	0.0008 (8)
C15	0.0397 (8)	0.0541 (9)	0.0403 (8)	0.0025 (7)	0.0181 (6)	0.0028 (7)
C16	0.0604 (11)	0.0676 (12)	0.0499 (10)	0.0043 (9)	0.0225 (8)	−0.0059 (9)
C17	0.0737 (13)	0.0998 (17)	0.0458 (10)	0.0081 (12)	0.0249 (9)	−0.0129 (11)
C18	0.0731 (13)	0.1169 (19)	0.0388 (9)	0.0139 (13)	0.0258 (9)	0.0106 (11)
C19	0.0648 (11)	0.0863 (14)	0.0458 (10)	0.0096 (10)	0.0253 (8)	0.0206 (10)
C20	0.0419 (8)	0.0604 (10)	0.0409 (8)	0.0014 (7)	0.0195 (6)	0.0103 (7)
C21	0.0426 (8)	0.0447 (8)	0.0450 (8)	−0.0009 (6)	0.0198 (7)	0.0064 (7)

### Geometric parameters (Å, °)

**Table:**

C1—C21	1.503 (2)	C9—C14	1.387 (2)
C1—C2	1.526 (2)	C10—C11	1.381 (3)
C1—H1A	0.97	C10—H10	0.93
C1—H1B	0.97	C11—C12	1.377 (3)
C2—C5	1.523 (3)	C11—H11	0.93
C2—C4	1.525 (3)	C12—C13	1.375 (3)
C2—C3	1.532 (3)	C12—H12	0.93
C3—H3A	0.96	C13—C14	1.381 (2)
C3—H3B	0.96	C13—H13	0.93
C3—H3C	0.96	C14—H14	0.93
C4—H4A	0.96	C15—C16	1.416 (2)
C4—H4B	0.96	C15—C20	1.417 (2)
C4—H4C	0.96	C16—C17	1.364 (3)
C5—C6	1.499 (2)	C16—H16	0.93
C5—H5A	0.97	C17—C18	1.399 (3)
C5—H5B	0.97	C17—H17	0.93
C6—O1	1.212 (2)	C18—C19	1.357 (3)
C6—C7	1.499 (2)	C18—H18	0.93
C7—C8	1.384 (2)	C19—C20	1.415 (2)
C7—C21	1.430 (2)	C19—H19	0.93
C8—C15	1.430 (2)	C20—N1	1.362 (2)
C8—C9	1.494 (2)	C21—N1	1.320 (2)
C9—C10	1.386 (2)		
			
C21—C1—C2	113.98 (14)	C10—C9—C8	121.06 (15)
C21—C1—H1A	108.8	C14—C9—C8	119.80 (14)
C2—C1—H1A	108.8	C11—C10—C9	120.35 (18)
C21—C1—H1B	108.8	C11—C10—H10	119.8
C2—C1—H1B	108.8	C9—C10—H10	119.8
H1A—C1—H1B	107.7	C12—C11—C10	120.24 (17)
C5—C2—C4	110.65 (16)	C12—C11—H11	119.9
C5—C2—C1	106.81 (14)	C10—C11—H11	119.9
C4—C2—C1	110.65 (16)	C13—C12—C11	119.75 (17)
C5—C2—C3	109.63 (16)	C13—C12—H12	120.1
C4—C2—C3	109.35 (16)	C11—C12—H12	120.1
C1—C2—C3	109.72 (15)	C12—C13—C14	120.38 (18)
C2—C3—H3A	109.5	C12—C13—H13	119.8
C2—C3—H3B	109.5	C14—C13—H13	119.8
H3A—C3—H3B	109.5	C13—C14—C9	120.24 (16)
C2—C3—H3C	109.5	C13—C14—H14	119.9
H3A—C3—H3C	109.5	C9—C14—H14	119.9
H3B—C3—H3C	109.5	C16—C15—C20	118.51 (16)
C2—C4—H4A	109.5	C16—C15—C8	123.45 (16)
C2—C4—H4B	109.5	C20—C15—C8	118.04 (15)
H4A—C4—H4B	109.5	C17—C16—C15	120.7 (2)
C2—C4—H4C	109.5	C17—C16—H16	119.7
H4A—C4—H4C	109.5	C15—C16—H16	119.7
H4B—C4—H4C	109.5	C16—C17—C18	120.5 (2)
C6—C5—C2	115.93 (15)	C16—C17—H17	119.7
C6—C5—H5A	108.3	C18—C17—H17	119.7
C2—C5—H5A	108.3	C19—C18—C17	120.46 (19)
C6—C5—H5B	108.3	C19—C18—H18	119.8
C2—C5—H5B	108.3	C17—C18—H18	119.8
H5A—C5—H5B	107.4	C18—C19—C20	120.8 (2)
O1—C6—C5	119.46 (16)	C18—C19—H19	119.6
O1—C6—C7	122.07 (16)	C20—C19—H19	119.6
C5—C6—C7	118.41 (15)	N1—C20—C19	117.99 (17)
C8—C7—C21	119.22 (14)	N1—C20—C15	123.00 (14)
C8—C7—C6	122.53 (14)	C19—C20—C15	119.00 (17)
C21—C7—C6	118.23 (14)	N1—C21—C7	123.40 (15)
C7—C8—C15	118.13 (14)	N1—C21—C1	115.98 (14)
C7—C8—C9	123.98 (14)	C7—C21—C1	120.62 (14)
C15—C8—C9	117.88 (14)	C21—N1—C20	118.11 (14)
C10—C9—C14	119.03 (15)		
			
C21—C1—C2—C5	−55.2 (2)	C8—C9—C14—C13	176.85 (16)
C21—C1—C2—C4	65.3 (2)	C7—C8—C15—C16	177.41 (15)
C21—C1—C2—C3	−173.95 (17)	C9—C8—C15—C16	−1.8 (2)
C4—C2—C5—C6	−67.6 (2)	C7—C8—C15—C20	−1.9 (2)
C1—C2—C5—C6	52.9 (2)	C9—C8—C15—C20	178.84 (14)
C3—C2—C5—C6	171.73 (16)	C20—C15—C16—C17	−1.7 (3)
C2—C5—C6—O1	158.93 (18)	C8—C15—C16—C17	178.98 (17)
C2—C5—C6—C7	−23.8 (2)	C15—C16—C17—C18	−0.1 (3)
O1—C6—C7—C8	−7.4 (3)	C16—C17—C18—C19	1.0 (3)
C5—C6—C7—C8	175.35 (16)	C17—C18—C19—C20	0.1 (3)
O1—C6—C7—C21	171.17 (18)	C18—C19—C20—N1	176.85 (18)
C5—C6—C7—C21	−6.1 (2)	C18—C19—C20—C15	−1.9 (3)
C21—C7—C8—C15	−0.8 (2)	C16—C15—C20—N1	−176.02 (16)
C6—C7—C8—C15	177.79 (14)	C8—C15—C20—N1	3.4 (2)
C21—C7—C8—C9	178.40 (14)	C16—C15—C20—C19	2.7 (2)
C6—C7—C8—C9	−3.0 (2)	C8—C15—C20—C19	−177.96 (15)
C7—C8—C9—C10	−76.1 (2)	C8—C7—C21—N1	2.5 (2)
C15—C8—C9—C10	103.06 (18)	C6—C7—C21—N1	−176.10 (15)
C7—C8—C9—C14	107.68 (19)	C8—C7—C21—C1	−178.28 (15)
C15—C8—C9—C14	−73.2 (2)	C6—C7—C21—C1	3.1 (2)
C14—C9—C10—C11	0.3 (3)	C2—C1—C21—N1	−151.45 (15)
C8—C9—C10—C11	−175.97 (17)	C2—C1—C21—C7	29.3 (2)
C9—C10—C11—C12	−1.0 (3)	C7—C21—N1—C20	−1.2 (2)
C10—C11—C12—C13	0.9 (3)	C1—C21—N1—C20	179.53 (15)
C11—C12—C13—C14	−0.1 (3)	C19—C20—N1—C21	179.55 (15)
C12—C13—C14—C9	−0.7 (3)	C15—C20—N1—C21	−1.8 (2)
C10—C9—C14—C13	0.6 (3)		

### Hydrogen-bond geometry (Å, °)

**Table:**

*D*—H···*A*	*D*—H	H···*A*	*D*···*A*	*D*—H···*A*
C11—H11···Cg1^i^	0.93	3.20	3.814 (3)	126

Symmetry codes: (i) *x*+1/2, *y*+3/2, *z*.
